# ^68^Ga-PSMA PET-CT Imaging of Metastatic Adenoid Cystic Carcinoma

**DOI:** 10.1007/s13139-016-0445-6

**Published:** 2016-08-16

**Authors:** Bart de Keizer, Gerard C. Krijger, F. Tessa Ververs, Robert J. J. van Es, Remco de Bree, Stefan Willems

**Affiliations:** 1 Department of nuclear medicine and radiology, UMC Utrecht, room E01.132, Heidelberglaan 100, 3584 CX Utrecht, Netherlands; 2 Department of clinical pharmacy, UMC Utrecht, Utrecht, Netherlands; 3 Department of head and neck surgical oncology, UMC Utrecht, Utrecht, Netherlands; 4 Department of pathology, UMC Utrecht, Utrecht, Netherlands

**Keywords:** Adenoid Cystic, Carcinoma, (68)Ga-PSMA, Immunohistochemistry, PET-CT

## Abstract

A patient with a history of adenoid cystic carcinoma of the nasal cavity presented himself with bone pain and an elevated PSA level. On suspicion of metastatic prostate cancer a ^68^Ga-PSMA PET-CT was performed. The PET-CT showed numerous lung and non-sclerotic bone metastasis. Biopsy of a bone metastasis was performed and pathology showed adenoid cystic carcinoma instead of prostate cancer. Immunohistochemical PSMA staining of the primary tumour showed intense PSMA expression in adenoid cystic carcinoma tumour cells. Because of the high PSMA expression of adenoid cystic carcinoma, ^68^Ga-PSMA PET-CT might be a promising imaging modality for this malignancy.

**Fig. 1:**
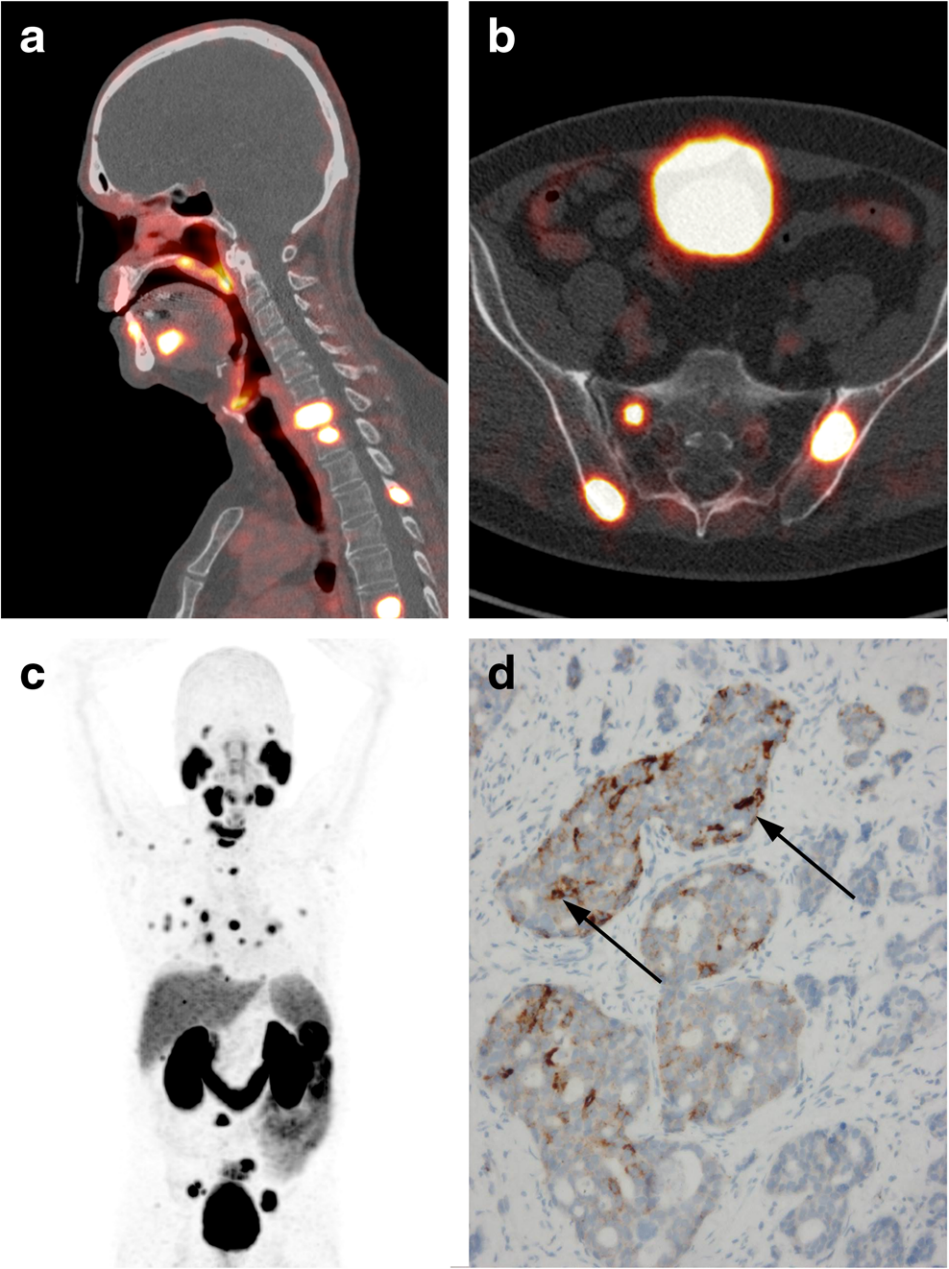
Sagittal (**a**) and axial (**b**) fused PET-CT images and coronal maximum intensity projection of ^68^GA-PSMA PET (**c**) showing intense PSMA binding in adenoid cystic carcinoma metastases. Immunohistochemistry of the primary tumour showed intense PSMA expression mainly in adenoid cystic carcinoma tumour cells (**d**, arrows)

